# The Phylogeny and Biological Function of Gastric Juice—Microbiological Consequences of Removing Gastric Acid

**DOI:** 10.3390/ijms20236031

**Published:** 2019-11-29

**Authors:** Tom C. Martinsen, Reidar Fossmark, Helge L. Waldum

**Affiliations:** 1 Department of Clinical and Molecular Medicine, Faculty of Medicine and Health Sciences, Norwegian University of Science and Technology (NTNU), 7006 Trondheim, Norway; reidar.fossmark@ntnu.no (R.F.); helge.waldum@ntnu.no (H.L.W.); 2 Department of Gastroenterology and Hepatology, Clinic of Internal Medicine, St Olav’s Hospital—Trondheim University Hospital, 7006 Trondheim, Norway

**Keywords:** gastric juice, gastric acidity, hypochlorhydria, proton pump inhibitors, gastointestinal infections, pneumonia, adverse effects, microbiome

## Abstract

Gastric juice is a unique combination of hydrochloric acid (HCl), lipase, and pepsin. Acidic gastric juice is found in all vertebrates, and its main function is to inactivate microorganisms. The phylogenetic preservation of this energy-consuming and, at times, hazardous function (acid-related diseases) reflects its biological importance. Proton pump inhibitors (PPIs) are one of the most widely used drugs in the world. Due to the reduced prevalence of *Helicobacter pylori* infection as well as the increased use of inhibitors of gastric acid secretion, the latter has become the most important cause of gastric hypoacidity. In the present manuscript, we review the microbiological consequences of removing gastric acidity. The resulting susceptibility to infections has not been studied extensively, and focus has mainly been restricted to bacterial and parasitic agents only. The strongest evidence concerning the relationship between hypochlorhydria and predisposition to infections relates to bacterial infections affecting the gastrointestinal tract. However, several other clinical settings with increased susceptibility to infections due to inhibited gastric acidity are discussed. We also discuss the impact of hypochlorhydria on the gut microbiome.

## 1. Introduction

### 1.1. The Biological Function of Gastric Juice

Gastric juice is a unique combination of hydrochloric acid (HCl), lipase, and pepsin. Its main function is to inactivate swallowed microorganisms, thereby inhibiting infectious agents from reaching the intestine. The mucosa in the mouth and the esophagus is squamous epithelium which, like the skin, could be presumed to protect against infection. On the other hand, the epithelium in the stomach and gut is of the simple columnar type, which would be expected to be more easily penetrated by infectious agents. The gastric juice is thus the first line of defense against infection throughout the gastrointestinal tract.

In addition to immunological mechanisms, intestinal motility, bile and pancreatic secretion, and intestinal microflora, the gastric juice constitutes an important factor in the defence against invasion of the gut by microorganisms [1]. These functions complement each other, exemplified by delayed gastric emptying and vomiting during upper gastrointestinal infections, thereby restricting further entry of pathogenic agents into the gastrointestinal tract. Moreover, delayed gastric emptying enhances the antimicrobial effect of the gastric juice.

The concept of “a gastric bactericidal barrier” has been discussed for a century [2,3].

In 1934 Hurst stated that “the Services would have saved much invaliding if men with achlorhydria were not sent to the tropics” on the basis of observations that dysentery and similar infections occurred more commonly in individuals with impaired gastric acid secretion [4]. The inactivation of microorganisms (not just restricted to bacteria as in Bartle and Hawkins “gastric bactericidal barrier”) [2] is suggested to be primarily pH–hydrochloric acid dependent, because the other constituents of gastric juice seem to have little effect on the destruction of infective agents [5,6]. However, to separate the effects of acid and pepsin seems hardly possible, because the enzyme’s activity always accompanies low pH and vice versa.

In addition to the killing of microorganisms, the acidic juice denatures proteins and augments absorption of dietary calcium and iron.

### 1.2. The Phylogeny and Physiology of Gastric Acid Secretion

Gastric acid secretion is a phylogenetically old function, probably first developed in cartilaginous fish more than 400 million years ago [7]. Although a highly energy consuming and at times hazardous function (acid-related diseases such as peptic ulcers and gastro-esophageal reflux disease (GERD), secretion of acidic gastric juice is preserved in fish, amphibians, reptiles, birds, and mammals, implying that it is important and evolutionarily advantageous [8].

Gastric HCl is secreted from the highly specialized parietal cells located in the corpus of the stomach, generating a H^+^ concentration in the gastric juice that is 3 million times greater than that in blood and tissue. The process is controlled by a complex system of endocrine cells and neurons.

Gastrin is the main hormone regulating gastric acid secretion, and is phylogenetically older than gastric acidity [8]. The gastrin family has been traced back to the *Ciona intestinalis* [9,10], a representative of the protochodrates that represents a key point in the phylogenetic transition to vertebrates. Indicative of a function of gastrin, gastric acid secretion appears to have developed concomitantly in the cartilagenous fish [8], in which also the so-called gastric enterochromaffin-like (ECL) cell is recognized as the target cell for gastrin in the oxyntic mucosa. While gastrin is well conserved in all vertebrates, a major structural change of gastrin accompanied the transition to mammals, giving rise to quite different types of gastrin (non-mammalian and mammalian) [7]. Gastrin is synthesized and released into the bloodstream by the gastrin cells in the antrum of the stomach, an ‘‘open type’’ of endocrine cell with microvilli on the apical membrane of its luminal surface allowing the cell to sense the luminal content, including its H^+^ concentration [11,12].

H^+^ ions have an inhibitory effect on gastrin cell activity at a pH below 4; an increase in gastric pH leads to a decrease in gastrin cell inhibition and an increase in gastrin release [13].

In addition to gastrin, histamine and acetylcholine are physiologically important signals; however, the way they all interact to stimulate gastric acid secretion is still being debated. Nevertheless, it seems generally accepted that gastrin acts by releasing histamine from the ECL cells by activating gastrin receptors [14,15,16], and that histamine stimulates the histamine-2 receptor on the parietal cell [11,12].

At a pH below 4 the gastric juice has a rapid bactericidal effect, in which exogenous bacteria introduced into the stomach are usually destroyed within 15 min. The bactericidal effect is reduced at a pH above 4.0 [6], and anything that raises the intragastric pH above 4 will allow bacterial overgrowth [5]. Interestingly, in healthy subjects, gastric acidity is maintained with a pH below 4.0 [17,18], which is similar to the pH level at which the inhibition of gastrin release from the antral gastrin cells stops [13].

Hence, the physiological apparatus of gastric acid secretion is designed to keep gastric pH below 4, the pH level essential to kill potential microbiological invaders [19].

### 1.3. PPI Induced Gastric Hypochlorhydria

Before the entry of drugs inhibiting gastric acid secretion, acquired conditions of hypochlorhydria such as chronic atrophic gastritis and malnutrition were the most common causes of gastric hypoacidity.

Due to the reduced prevalence of *Helicobacter pylori* infection, as well as the increased use of antisecretagous drugs, the latter has become the dominating cause of gastric hypo/achlorhydria. Proton pump inhibitors (PPIs) inhibit the gastric H^+^K^+^ATPase (the proton pump) by binding covalently and irreversibly to the alpha subunit. Both basal and stimulated secretion of gastric acid is inhibited, independent of the nature of parietal cell stimulation, and all PPIs are more or less similar in efficacy and potency.

PPIs are among the top 10 most widely used drugs in the world, and recently became the second largest drug in terms of revenue in the USA [20,21]. The main indications for PPIs are acid-related conditions such as gastroesophageal reflux disease and peptic ulcer, but they are also frequently prescribed for management of dyspepsia, as part of *Helicobacter pylori* eradication therapy, and for prevention of peptic ulcer bleeding in high-risk patients on aspirin and/or non-steroidal anti-inflammatory drugs. Unfortunately, numerous studies in Western populations have documented prevalent PPI prescription and use without evidence-based indication [22,23,24,25,26]; hence, in many populations, patients without indication may be the largest group of users.

## 2. Microbiological Consequences of Removing Gastric Acid

From a biological point of view, it seems likely that removal of such a phylogenetically preserved function as gastric acid secretion found in all vertebrates will have consequences. Furthermore, in contrast to most other drugs that modify or normalize physiological mechanisms and/or parameters, such as, for instance, antihypertensives, gastric acid secretion is nearly eradicated by the widely used antisecretagogues.

Although gastric hypo/achlorhydria is common due to chronic atrophic gastritis or use of inhibitors of gastric acid secretion, the resulting susceptibility to infection has not been studied extensively. Admittedly, since Bartle and Hawkins [2], it has repeatedly been demonstrated that reduction of gastric acid secretion predisposes to infection with a variety of microbiological organisms [27,28,29,30,31,32,33,34]. However, remarkably few high quality experimental or epidemiological studies have been performed and the majority of existing studies are rather old (although an increasing number were published in the last decade), and mainly restricted to bacterial and parasitic agents only.

Moreover, one of the main problems with the studies that have aimed to reveal an association between hypochlorhydria and increased susceptibility to microorganisms conducted before the entry of PPIs is that several infections themselves reduce gastric acid secretion in humans [1,30,35,36,37,38,39] and animals [1,30,40,41]. The mechanism underlying infection-evoked suppression of acid secretion is not well understood [1]. In addition, fever per se may inhibit gastric acid secretion in humans [42,43]. Therefore, experimental longitudinal (before, during, and after infection) studies to compensate for these biases are required. Finally, malnutrition is associated with hypo/achlorhydria [29,44], and the extent to which subclinical malnutrition affects gastric acidity is unknown [27]. Malnutrition also impairs other factors in the intestinal defense system. Consequently, the combination of malnutrition and hypo/achlorhydria puts individuals at high risk of enteric infections, facing a vicious circle (malnutrition and hypo/achlorhydria).

Surprisingly, very few studies have focused on the effect of reduced gastric acidity on the susceptibility to viral infections [27,32,34,45]. A hallmark of enteroviruses is their stability at low pH. Accordingly, they will reach the gut and multiply there, gaining access to the blood or the central nervous system via peripheral nerves [46]. Still, it seems reasonable to assume that many viruses are sensitive to the uniquely low pH in the gastric juice [45] which, theoretically, would predispose patients with hypo/achlorhydria to viral infections. Indeed, rapid inactivation of rotaviruses by exposure to acidic buffer and acidic gastric juice at pH 2, but not at pH 4, has been reported [47]. It has been shown that influenza viruses infect and persist in gastric mucosa in patients receiving antisecretagogues [48]. Indeed, community-acquired respiratory infections (see Section 2.3), which may be viral in origin, seem to be more common in users of acid-suppressing drugs.

In addition, there have been few studies concerning the role of gastric acidity in protection against fungal infections. Although systemic candida infections have been reported in immunocompromised patients treated with histamine- 2 receptor blockers (H2RBs) [49], the majority of existing reports concerns affection of the gastrointestinal tract (see Section 2.1.3).

### 2.1. Hypochlorhydria and Infections of the Gastrointestinal Tract

The strongest evidence concerning the relationship between hypo/achlorhydria and predisposition to infections relates to infections affecting the gastrointestinal tract (Table 1).

In a retrospective study, Nwokolo and co-workers [50] found that patients taking acid-suppressing drugs (H2RBs or PPIs) appear to have approximately three times the risk of bacterial diarrhea than non-recipients. Moreover, in a case–control study of 6414 cases and 50,000 controls, a RR of 2.9 was reported between PPI use and bacterial gastroenteritis [51]. Cobelens et al. [52] found an increased risk of diarrhea in travellers taking antacid or H2RBs. The International Society of Travel Medicine has identified PPI use as a risk factor for travellers’ diarrhea, and suggests daily antibiotics to prevent illness in PPI users during travel to high-risk areas [53]. In a case–control study of 170,000 ever-users of acid-supressing drugs (H2RBs or PPIs) [54] identifying 374 bacterial gastroenteritis and 2000 controls, the authors concluded that they did not find any important association between the use of acid-suppressing drugs and risk of bacterial gastroenteritis in general. However, the RR among omeprazole users for 2 months was 1.6. The study was nested within a cohort of long-term users of anti-secretory drugs; consequently, it is possible that the study design selected out subjects with previous gastroenteritis while on these drugs [55].

In the recent placebo-controlled, randomized COMPASS trial [56] including 17,598 participants with stable cardiovascular disease and peripheral artery disease, patients randomized to use of the PPI pantoprazole had an increased risk of self-reported enteric infections (*Clostridium difficile* infection (CDI) not included) with an odds ratio (OR) of 1.33 during follow up for a median of 3.01 years. The number needed to assume harm for enteric infections was 301. In a prospective study performed in pediatric patients, it was demonstrated that the use of inhibitors of gastric acid secretion (H2RBs or PPIs) was associated with an increased risk of acute gastroenteritis (OR 3.58) [57].

#### 2.1.1. Bacterial Enteric Infections

The strongest evidence in favour of the view that hypo/achlorhydric patients are susceptible to bacterial infections relates to non-typhoid salmonelloses and cholera, both bacteria known to be acid-sensitive. Impaired acid secretion increases both the frequency and severity of these infections [27,29,30,58,59,60,61,62]. However, studies on the impact of PPI as a risk factor are rather few. Despite the widely held belief that gastric acid serves as a barrier to bacterial pathogens, the experimental data to support this hypothesis is rather sparse. However, some experimental studies on different bacteria are presented here before the clinical observational/epidemiological studies concerning eight specific bacterial agents.

##### Experimental Studies of Bacterial Infections due to Hypo/Achlorhydria

In 1885, Koch demonstrated *Cholera* infection in guinea pigs if the pathogens were delivered with bicarbonate [63]. Almost a century later, Hornick’s group applied the “bicarbonate model” in humans, showing that when sodium bicarbonate was given with live *Cholera vibrios* to volunteers, the infecting dose was lowered from 10^8^ to 10^4^ organisms [58,64]. Further experimental studies on volunteers demonstrated that diarrhea after ingestion of *C. vibrios* and sodium bicarbonate was closely correlated to basal gastric acidity [65].

In an adult volunteer challenge study published by DuPont et al. in 1971 [66], invasive *Escherichia coli* produced diarrhea only when the organisms were administered after neutralising gastric acid with sodium bicarbonate, showing the importance of reducing gastric acidity in the development of diarrhea caused by this agent. In 1972, Gianella et al. [5] found that *Salmonella paratyphi* and *Salmonella enteritidis* did not survive at pH < 3, whereas at pH > 4, no reduction in bacterial count was observed. The same paper showed decreased survival of diarrhea-producing *Escherichia coli* at pH < 3.5, and also documented that the minimum pH for proliferation is 4.4 [67]. One study in mice has also illustrated the protective role of gastric juice against *Salmonella* infections [68]. Moreover, *Campylobacter jejuni* has been found to be more susceptible to gastric acid than *Salmonella* [69]. Waterman and Small [70] recovered very few colonies of *Campylbacter jejuni* upon exposure to a relative high pH of 4 and 5. However, *Campylbacter jejuni* showed increased survival at pH 6, a pH possible with high doses of PPIs. Subsequently, in an important study by Tennant et al. [71], mice that were constitutively hypochlorhydric due to a mutation in a gastric H^+^K^+^ATPase gene were used as a model to quantify the effectiveness of gastric acid in mediating resistance to infection with ingested bacteria (*Yersinia enterocolitica*, *Salmonella enterica serovar Typhimurium*, *Citrobacter rodentium*, or *Clostridium perfringens* cells or spores). Before performing the animal experiments, they investigated the survival of *Yersinia enterocolitica 8081*, *Yersinia enterocolitica 8081u*, *Salmonella enterica serovar Typhimurium*, and *Clostridium rodentium* in phosphate-buffered saline at various pHs. The results supported previous findings [72,73] that *Yersinia enterocolitica* is highly acid-resistant, and that this phenotype is mediated by the ability of this organism to produce urease. The results for *Salmonella enterica serovar Typhimurium* supported those of Gorden and Small [74], who found that *Salmonella* species are unable to survive at pH 2.5 for 2 h. The acid resistance of *Clostridium rodentium* (not tested previously), was found to be slightly lower than *Salmonella enterica serovar Typhimurium*. In the mouse studies, significantly greater numbers of *Yersinia*, *Salmonella*, and *Citrobacter* cells and *Clostridium* spores survived in hypochlorhydric mice, resulting in reduced median infectious doses. This was in concordance with Sun et al. [75], who performed controlled experiments which showed that non-pathogenic *Escherichia coli* survives better in hypochlorhydric gastrin-deficient mice than in wild-type mice. Finally, experiments involving intraperitoneal infection (inoculation of mice by a route that bypassed the stomach) or infection of mice treated with antacids indicated that the increased sensitivity of hypochlorhydric mice to infection was entirely due to the absence of stomach acid [71]. In general, *Listeria* is vulnerable to a pH ≤ 2, with increased survival at pH ≥ 5 [76,77]. However, different strains of *Listeria* show various degrees of susceptibility to gastric acid. Moreover, in a rat model, rats pretreated with cimetidine, a H2RB, showed a significantly lowered infective dose of virulent *Listeria monocytogenes* [78].

##### Specific Bacterial Agents 

**(1) *Non-typhoid salmonellosis*:** Before the entry of antisecretagous drugs, the best evidence for increased susceptibility to enteric infections following hypo/achlorhydria was the increased occurrence [79,80,81,82,83,84] and severity [80,85] of salmonellosis following gastric surgery inhibiting gastric acid secretion. The indications for gastric resection/gastrectomy in these studies were primarily peptic ulcer disease or gastric malignancy. Since then, several studies have identified H2RBs [59,60,62] and PPIs [31,61] as risk factors for non-typhoid salmonelloses. In a case–control study of 360 cases and 3119 controls [86], the multivariate OR when using PPI compared to not using PPI were found to be 4.2 for *Salmonella enteritidis* and 8.3 for *Salmonella typhimurium*. In another case–control study of 573 cases and 3409 controls [51] the multivariate OR was similar at 4.3.

**(2) *Cholera*:***Vibrio cholerae*, the cause of cholera, an important cause of dehydrating diarrhea in endemic areas of the developing countries, is very acid-sensitive [87]. In addition to conclusions from bicarbonate models of Koch [63] and Hornick [58] (see above) it was found that hypo/achlorhydria was over-represented among patients in Pakistan with cholera [88]. A study of an outbreak in Thailand described an increased association of antacid use among cholera cases [89]. However, there is a lack of studies exploring the explicit impact of PPI use on the susceptibility of *Cholera* infection.

**(3) *Campylobacter jejuni*:** Infections with *Campylobacter* spp have been linked to hypo/achlorhydria in general [90]. The first case–control study on *Campylobacter* gastroenteritis where PPI was recognized as a risk factor was published in 1996 [91], and showed a 10-fold increased risk for infection. Since then, three more case–control studies have reported an association between *Campylobacter* diarrhea and PPI use, with adjusted RR ranging from 3.5 to 4.5 [92,93,94].

**(4) Diarrheagenic *Escherichia coli:****Escherichia coli* has also been associated with hypo/achlorhydria in humans [87,95]; thus, a high gastric pH induced by PPI may facilitate the pathogenesis of *Esherichia coli* diarrhea. There are some experimental data supporting this [5,66,67,75] (see above). However, there has been a lack of studies attempting to determine the association of PPI use and susceptibility to the various diarrhea-producing *Escherichia coli* strains.

**(5) *Clostridium difficile*:***Clostridium difficile* is the most common cause of nosocomial diarrhea and the main cause of colitis in hospitalized patients, thus placing a high burden on patients and the healthcare system [96,97,98], ranging in severity from mild diarrhea to fulminant colitis and even death [99]. Moreover, many patients suffer from recurrent *Clostridium difficile* infection (rCDI). The association between the use of PPIs and the risk for CDI has been better studied than all other gastrointestinal infections. There are two forms of *Clostridium difficile*: The acid-resistant spore form and the vegetative toxin-producing, acid-sensitive form that typically fails to survive normal gastric acidity [100,101,102]. Thus, spores are considered to be the major form for transmission, although the vegetative form is by far the most abundant in stool of infected individuals [100]. However, under favourable conditions, such as high gastric pH induced by PPI, both increased survival and propagation of the vegetative forms, and enhanced conversion of spores to vegetative forms, promotes the development of CDI [31,100]. Antibiotics are clearly the most common cause of CDI, altering the flora and providing an opportunity for growth of *Clostridium difficile*. However, the combination of antibiotics and PPI, a common clinical setting, appears to work together in an additive fashion for increasing the susceptibility to CDI. Since the first study addressing PPI as a risk factor for CDI by Cunningham et al. in 2003 [103], dozens of case–control and cohort studies have been published focusing upon this issue [31,98]. The majority of the studies have examined the association between PPI and CDI in hospitalized patients, including patients in intensive care units (ICUs), and some in community-associated patients. Unfortunately, studies in the early 2000s demonstrated a high degree of heterogeneity and a high percentage of negative results [98]. Since 2011, the overall association between PPI use and risk for developing CDI has remained relatively stable within an effect size between OR of 1.20 and 1.26 [98]. A recent pooled analysis of 50 studies showed an overall OR of 1.26 [98]. The relative risk seems to be higher for hospital-acquired CDI (OR 1.29) than community-associated CDI (OR 1.17); furthermore, the risk seems to be higher in ICUs (OR 1.43) compared to general wards (1.29). Finally, several retrospective cohort studies of hospitalized patients have revealed an association between PPI use and development of rCDI, showing relative risk of 1.4–4.2 [104,105,106]. While studies have been somewhat inconsistent, a 2012 meta-analysis of 42 studies found that PPI use was associated with an increased risk for initial and rCDI [107]. This led the US Food and Drug Administration to issue a drug safety warning in 2012 regarding this association. In the recent COMPASS trial [56], the rate for self-reported *Clostridium difficile* infection was approximately twice as high in the pantoprazole vs. the placebo group, although there were only 13 events, so this difference was not statistically significant (*p* = 0.18). Beyond this, we found no randomized, placebo-controlled clinical trials that could support causality between an increased risk of CDI and PPI use.

**(6)Shigellosis:** Shigellosis is a common form of dysenteric colonic infection leading to diarrhea. Neutralization of the gastric content with bicarbonate enhances the frequency of *Shigella* in the stool [108]. However, one study stated that shigellosis occurs equally often in people with normal gastric acidity, and that gastric acid does not influence the susceptibility to *Shigella* infection [87]. The reported acid resistance of *Shigella* [67,109] supports the latter conclusion. In contrast to many other bacterial organisms, strains of *Shigella* can survive exposure to acid in the stomach [108]. There have been no studies evaluating the association between shigellosis and PPI use. However, it is unlikely that PPI use would increase the risk of infection by *Shigella* strains due to their low inoculum requirements and relative acid resistance.

**(7) Listeriosis:** Strains of *Listeria* have been isolated from the stools of patients receiving H2RBs [110]. Further, listeriosis was associated with the use of antacids and H2RBs in a retrospective epidemiological study by Ho et al. [111]. They found that patients receiving antacids or H2RBs were more likely than controls to be infected during a foodborne outbreak of hospital-acquired listeriosis. Subsequently, several studies have supported the association between gastric hypoacidity and listeriosis [95]. It is likely that people using PPIs are more susceptible to this organism, which is capable of causing fatal disease in immunocompromised patients or in the elderly. Numerous population-based studies have found an increased risk of *Listeria* infection in PPI users [112,113,114,115]. In a Danish case–control study including 721 cases of listeriosis compared to 34,800 controls [116], the adjusted OR for current use of PPIs and development of listeriosis was 2.81. This is of concern, as increasing incidence of listeriosis has been registered in several of these countries.

**(8)Brucellosis:** Brucella infections have been reported after use of both antacids [117] and H2RBs [118,119]. As far as we know, no further studies have been published concerning the potential increased risk of brucellosis induced by hypo/achlorhydria.

#### 2.1.2. Parasitic Enteric Infections

The strongest evidence for an association between hypochlorhydria and increased risk for parasitic infections is for giardiasis and strongyloidiasis. However, there is a lack of studies to support a definite conclusion, especially concerning drug-induced hypo/achlorhydria. The use of H2RBs has been associated with *Strongyloides* infections [120], especially in immunosuppressed patients [121,122]. Moreover, there is a report of two patients developing gastric giardiasis after short term treatment with PPI [123]. However, the clinical consequences of drug-induced impaired gastric acidity and a suggested increased susceptibility to infection with *Strongyloides* and *Giardia* are still not settled. Interestingly, rats pretreated with an H2RB can be infected with *Entamoeba histolytica* by the oral route, unlike untreated rats [124]. This suggests that gastric acid may be an important defence mechanism against this protozoa. In contrast, patients taking a PPI were statistically less likely to have intestinal protozoa reported on a stool ova and parasite examination compared with those not taking a PPI [125]. Laboratory studies have shown that PPIs have antiprotozoal activity [126,127,128,129].

#### 2.1.3. Fungal Gastrointestinal Tract Infections

Localized *Candida* infections of the oesophagus have repeatedly been linked to use of antisecretagous drugs [130,131,132,133]. A recent retrospective study of 55,314 Koreans revealed that use of gastric acid suppression therapy is an independent risk factor (OR 5.11) [134].

In contrast, a large (80,210 patients) Japanese study failed to find such an association [135]. Growth of *Candida alibicans* in the stomach has been noted following vagal denervation [136], partial gastrectomy [137], H2RB therapy [138,139], and during treatment with PPIs [139,140]. Moreover, localized *Candida* infections of duodenum [141] and the small intestine [142] have been reported in association with the use of antisecretagogues.

### 2.2. Hypochlorhydria and the Gut Microbiome

The gut microbiome plays an important role in enteric infections [143,144,145,146], and gut microbiota can resist or promote microbial colonization of the gut [143,144,145]. The microbiome is being intensively studied in various diseases and conditions including inflammatory bowel disease (IBD), irritable bowel syndrome (IBS), obesity, old age, non-alcoholic steatohepatitis, and non-alcoholic fatty liver disease. PPI users are over-represented in these groups, as they are more likely to have gastrointestinal complaints or experience GERD, whether due to their health condition or their associated lifestyle. The increased incidence of enteric infections in PPI users and the importance of the gut microbiome composition in the development of these infections make it rational to investigate the influence of PPI use on the gut microbiome. Moreover, gut microbiota can resist or promote colonisation of *Clostridium difficile* and other enteric infections through mechanisms that either directly inhibit bacterial growth or enhance immune system infections [143,144,145,146]. In a study of the gut microbiome composition of 1815 individuals, 211 of whom used PPI [147], PPIs were shown to be associated with a significant decrease in Shannon’s diversity and with changes in 20% of the bacterial taxa. Multiple oral bacteria were over-represented in the fecal microbiome of PPI users. Further, a significant increase in bacteria was observed in PPI users: Genera *Enterococcus*, *Streptococcus*, *Staphylococcus*, and the potentially pathogenic species of *Escherichia coli*. The authors concluded that the differences between PPI users and non-users were consistently associated with changes towards a less healthy gut microbiome. These differences are in line with known changes that predispose to CDI and could potentially explain the increased risk of enteric infections in PPI users [147]. On a population level, the effects of PPI on the microbiome are more prominent than the effects of antibiotics or other commonly used drugs [148]. Given the widespread use of PPIs, the morbidity and mortality associated with enteric infections, and the increasing number of studies investigating the microbiome, healthcare practitioners and microbiome researchers should be fully aware of the influence of PPI on the gut microbiome, and future microbiome studies in humans should always take the effect of PPI on the gut microbiome into account. Moreover, in a study of fecal samples of 1827 healthy twins [149], a significant association was demonstrated between the composition of the gut microbiota and PPI use. There was a significantly lower abundance of gut commensals and lower microbial diversity in PPI users, with an associated significant increase in the abundance of oral and upper gastrointestinal tract commensals. The most striking association was an increase in Lactobacillales, particularly Streptococcaceae, in PPI users. The authors concluded that the observed alterations to the gut microbiota with PPI use might be responsible for the observed increases in infection risk, and therefore might provide targets for research to reduce these risks. The potential consequences of these changes are motivation for caution against unnecessary provision of PPIs. In a prospective study by Reveles et al. [150] of healthy older adults (age ≥ 60 years), participants (24 subjects) provided a stool sample at baseline, completed a 14 day course of omeprazole (a PPI) at 20 mg daily, and then provided a follow-up stool sample. As shown in previous studies, PPI use had impact on the microbiome. Pre-PPI samples had significantly higher relative abundance of the phylum Actinobacteria and the families Lachnospiraceae, Erysipelotrichaceae and Bifidobacteriaceae. Post-PPI samples had significantly higher abundance of Streptococcaceae, in accordance with previous studies by Imhann et al. [147] and Jackson et al. [149]. In fact, decreased Bifidobacterium found by Imhann et al. [147] and Reveles et al. [150] was associated with CDI [151], whereas supplementation with Bifidobacterium seems to reduce the risk of developing CDI in humans [152]. Moreover, the abundance of Streptococcaceae is increased in CDI, while Lachnospiraceae is reduced compared with healthy controls [153]. These findings, in addition to those of Imhann et al. [147] and Jackson et al. [149], underline the potential impact of PPIs on human health through alteration of the gut microbiota and the need to decrease inappropriate and unnecessary use of PPIs. The profound alterations seen in the gut microbiome could be linked to the increased risk of CDI and other enteric infections.

### 2.3. Hypochlorhydria and Respiratory Tract Infections

It has been hypothesized that the elevation of gastric pH caused by PPIs leads to overgrowth of bacteria in the upper gastrointestinal tract, which might subsequently move upwards and colonize the lower respiratory tract via gastroesophageal reflux and microaspiration.

The first large scale case–control study focusing on this issue, published by Laheij et al. in 2004 [154], demonstrated that the adjusted relative risk of community-acquired pneumonia among patients currently using PPIs was 1.89 in comparison with those who did not use PPIs. Since 2004, several studies [155] have been conducted to investigate this possible adverse effect of PPI therapy. Moreover, several meta-analyses of RCTs, cohort studies, and case–control studies have consistently demonstrated that PPI therapy might not only be associated with an increased risk of community-acquired pneumonia, but also hospital-acquired pneumonia [155,156,157,158]. The most recent meta-analysis of 58 studies, including 10 RCTs and 48 observational studies with a total of more than 7.5 million patients, found an increased risk of pneumonia among PPI users (OR 1.43) [155]. However, the authors emphasized that the heterogeneity was high; thus, protopathic bias or reverse causality, which occurs in case–control studies when a pharmaceutical agent is inadvertently prescribed for an early manifestation of a disease that has not yet been diagnostically detected [159], may have caused overestimation of an association [155]. Subgroup analysis indicated that studies that adopted a design to account for protopathic bias (as for non-steroid anti-inflammatory drug users, PPIs were prescribed to prevent ulcers or dyspepsia rather than for the early symptoms of pneumonia) did not show a significant association between PPI use and risk of pneumonia. In addition, the impact of publication bias and confounding by indication was discussed thoroughly [155]. In a prospective study performed in pediatric GERD-affected patients, it was demonstrated that the use of inhibitors of gastric acid secretion (H2RBs or PPIs) was associated with an increased risk of community-acquired pneumonia (OR 6.39) [57].

#### Risk of Pneumonia in Different Group of Patients

In several patient groups associations between use of antisecretagogues and increased risk of pneumonia have been reported (Table 2).

**(1) Patients in ICU units:** Initially, the presence of microbes in the stomach was associated with the occurrence of nosocomial respiratory infection, in particular ventilator-associated pneumonia in patients at ICUs [160,161,162]. During prolonged mechanical ventilation in combination with the use of acid-suppressive therapy, the upper airways and the stomach become colonized with pathogenic bacteria, which may gain access to the lower airways. Although the quality of evidence supporting the prophylactic use of proton-pump inhibitors as stress ulcer prophylaxis in the ICU is limited [163,164,165], patients undergoing intensive care are treated with acid antisecretagogues. Thus, they are among the most frequently used off-label medications in ICUs (they have not been approved by the Food and Drug Administration as prophylaxis for stress ulcers) [166]. Concerns have been raised about increased risk of pneumonia associated with this class of drugs, which may counterbalance their potential benefits. Initially, a number of papers [167,168,169] and a meta-analysis showed an increased risk of pneumonia in PPI- and H2RB-treated intensive care patients. However, recent studies and meta-analyses [170,171] have failed to confirm this.

In a Danish-led, international, multicenter, blinded, placebo-controlled, randomized trial [172], randomly assigned adults, who had been admitted to a ICU for an acute condition and who were at risk for gastrointestinal bleeding, received the proton-pump inhibitor pantoprazole or placebo daily during their ICU stay. A total of 3298 patients were enrolled; of those, 1645 were randomly assigned to the pantoprazole group and 1653 to the placebo group. The primary outcome was death by 90 days after randomization. The number of patients with infections and the percentage of days alive without life support within 90 days were similar in the two groups. These results were similar to those obtained in a recent network meta-analysis [173], in which no significant differences were found in the rates of death or infectious complications between patients receiving placebo or no prophylaxis and those receiving PPIs.

**(2) Dementia:** A retrospective cohort study of 786 dementia patients using PPI compared to an equal number of matched controls without PPI found an 89% increased risk of pneumonia [174].

**(3) Acute stroke:** A meta-analysis [175] of five retrospective cohort studies including hospital-acquired pneumonia of patients with acute stroke showed an increased risk of pneumonia in patients using antisecretagous drugs (unadjusted RR 4.65), particularly those exposed to PPI.

**(4) Type II diabetes mellitus:** A retrospective cohort study suggested that PPI use increased the risk of pneumonia in patients with type II diabetes mellitus [176], demonstrating a cumulative incidence of pneumonia in PPI users 11.4% higher than in controls (30.3% vs. 18.9%) after a 14 year follow up.

**(5) Older adults:** A study of a cohort of older adults (60 and older) in primary care, in which 75,050 patients who had been receiving PPI for 1 year or longer were compared to equal numbered controls not receiving PPI, demonstrated that the pneumonia risk in the second year of treatment was greater (prior event rate ratio adjusted hazard ratio (HR) = 1.82) with long-term PPI therapy [177]. The increased risk was independent of excess pneumonia rates immediately before first PPI receipt.

**(6) Cirrhotic patients:** In a retrospective study of US veterans with decompensated cirrhosis [178], new PPI users were found to have increased rate (1.75 times faster than non PPI users) of serious infections, of which 25% were pneumonias. The study was not designed explicitly to examine the risk of pneumonia. In a mortality study of cirrhotic patients with pneumonia, but not active gastrointestinal bleeding, PPIs were not associated with 30 day mortality [179]. However, the authors stated that prolonged PPI therapy may be associated with higher mortality.

**(7) GERD**: In a population-based cohort study from Taiwan including 15,715 GERD cases and an equal number of non-GERD matched controls, GERD patients exhibited a 48% higher risk of developing pneumonia than the non-GERD individuals within 6 years [180]. Crucially, GERD patients using PPIs for longer than 4 months had an increased risk of pneumonia compared to those who did not use or took PPIs for less than 4 months. The highest increase in risk was in patients younger than 40 years of age (HR 2.17).

### 2.4. Hypochlorhydira and Liver Cirrhosis

PPIs are used by 46–78% of patients with cirrhosis [181,182], so it is critical that the adverse effects of these drugs are clarified. The risk of pneumonia in this setting is discussed in Section 2.3.

#### Risk of Microbiological Complications in Patients with Liver Cirrhosis

**(1) Spontaneous bacterial peritonitis (SBP):** Bacterial translocation has been described as a key mechanism in SBP development. Small intestinal bacterial overgrowth potentially promotes bacterial translocation [183,184]. Thus, it has been speculated that chronic acid suppression by proton pump inhibitors (PPIs), which favours gastric and duodenal bacterial colonization, might contribute to small intestinal bacterial overgrowth and consequently increase the incidence of SBP. In both case–control and cohort studies, as well as meta-analyses, there have been conflicting reports regarding the role of acid-suppressive therapy in predisposing patients with cirrhosis to SBP. Of the four meta-analyses published since 2011 [185,186,187,188], each including 4–17 studies (case–control studies and cohort studies) and 772–8145 patients, three concluded [185,186,188] that there is an association between PPI and the risk of SBP, with OR from 2.17–3.15. In contrast, the most recent could not establish causality that PPI use increases the incidence of SBE [187]. There have been far fewer studies concerning mortality risk due to PPI in SBE patients; two recent studies reported increased long-term mortality risk in PPI users [189,190], whereas other authors fail to find such an association [187,191]. Finally, one study examined whether PPI use increases the risk for recurrent SBP in cirrhotic patients and concluded that PPI is not a risk factor for recurrent SBP [192].

**(2) Hepatic encephalopathy (HE):** HE is a devastating complication to cirrhosis associated with a poor quality of life, a high risk of recurrence, and a poor prognosis. Gut-derived nitrogenous substances are universally acknowledged to play a major role in the pathogenesis of HE. PPI use may contribute to the gut dysbiosis commonly found in cirrhotic patients [193,194,195], whereas altered gut microbiota could be associated with HE. It is therefore disconcerting that PPI was for the first time found to be associated with HE in a small case–control study of Asian patients with hepatitis B-related acute-on-chronic liver failure [196]. In a recent population-based case–control study from Taiwan [197], patients with cirrhosis and an occurrence of HE (n = 1166) were compared to an equal number of matched controls without HE in terms of PPI use. The adjusted OR for patients with cumulative defined daily doses (cDDDs) of PPIs of more than 365 was 3.01. All PPI users (defined as more than 30 cDDDs) had an increased risk for HE. Hence, the authors concluded that the use of PPI in patients with cirrhosis increases the risk for HE, and the risk increases with dose. In a study analysing data from three large, multicentre, randomized 1 year trials of satavaptan for ascite control in 865 patients with cirrhosis and ascites [198], the adjusted HR of HE for current PPI users versus current non-users was 1.36. For overt HE (grade 2–4), the adjusted HR was 1.88, supporting the idea that PPI is a risk factor for developing HE in cirrhotic patients with ascites.

### 2.5. Hypochlorhydria and Infections in the Liver and Biliary System

**(1) Liver abscess:** In a population-based case–control study including 958 cases of cryptogenic liver abscesses and 3832 matched controls [199], the adjusted OR associating current use of PPIs (prescription within the past 30 days) with cryptogenic live abscess was 4.7, and recent use of PPIs (prescription within 31–90 days) was 2.9. A dose–response relationship was apparent for cumulative doses of PPIs within 90 days, with the highest adjusted OR (6.5) among the patients receiving PPIs at more than 60 cumulative defined daily doses.

**(2) Cholangitis:** In a cohort study with 58,863 participants (4,212,003 person-years follow up) with at least one PPI prescription [200], 1834 developed cholangitis, giving an adjusted HR for incident cholangitis of 5.75–6.06. The risk was highest during PPI treatment and decreased gradually after PPI discontinuation.

**(3) Cholecytitis:** A population-based case–control study involving 3192 patients with cholecystitis and 12,768 controls [201] demonstrated, after adjusting for comorbidities (more frequent in the cholecystitis group), that the PPI users still had a 1.23-fold increased risk of cholecystitis compared with the PPI non-users. The authors hypothesized that PPIs reduce the bactericidal activity and allow pathogens to pass through the stomach to the duodenum, thereby increasing the risk of retrograding to the biliary system, and thus elevating the incidence of biliary tract infection, including acute cholecystitis. In the study by Imhann et al. [147], stool samples from PPI users showed a significant increase, compared to non-PPI users, in bacteria—genera *Enterococcus*, *Streptococcus*, *Staphylococcus*, and *Escherichia coli*—some of which are also common pathogens in acute cholecystitis, thus strengthening the hypothesis of increased risk of cholecystitis in PPI users.

### 2.6. Hypochlorhydria and CNS Infections

In a retrospective cohort study enrolling 16,241 PPI-using patients with CNS infections (ICD-9-CM codes 320–324) [202], the incidence of CNS infection in the PPI users, after adjusting for confounding factors, was 2.23-fold higher than in the equal number of PPI non-users.

#### Transmissible Spongiform Encephalopathies—Prion Diseases

Prion diseases may be transmitted via the gastrointestinal tract, as shown in kuru and variant Creutzfeldt–Jakob disease [203]. Prions are not recognized as foreign by the immune apparatus, and thus do not induce any inflammation [204]. Recently, several neurodegenerative conditions have been linked to prion-like propagation within the nervous system [205,206,207]. It is well known that prions are very resistant infectious agents, and that infectivity is preserved after exposure to highly acidic liquids [208]. Therefore, gastric juice was believed not to play any role in defense against prion infections. However, acid exposure studies did not take into consideration that the gastric juice is more than just an acid. The enzymes in the gastric juice, particularly pepsin, but also possibly lipase, might be expected to contribute to the destruction of the prions. We accordingly performed two animal studies: Initially, we introduced different amounts of scrapie agent intragastrically in control mice and in mice where acid secretion was reduced by the H2RB ranitidine. Mice pretreated with ranitidine developed scrapie encephalopathy more frequently than control animals when exposed to lower doses of scrapie agent [209]. In the second study, we inhibited gastric acid secretion with the help of the PPI omeprazole, raising the intragastric pH from median 1.2 to 5.3. Compared with control mice, mice dosed with omeprazole had more than double the frequency of encephalopathy after exposure to scrapie agent [210]. We therefore concluded that normal gastric juice may have a protective role in the defense against prion diseases. The reports that truncal vagotomy could be protective against Parkinson’s disease [211] and the association of PPIs with risk of dementia [212,213], (although some conflicting findings have been published [214,215]), support the possible role of the gut as a gateway for entrance of neurotropic pathogens to the brain.

## 3. Conclusions

The preservation of acidic gastric juice secretion during phylogenesis supports the biological importance of this highly energy-consuming function developed to inactivate ingested microorganisms. Iatrogenic gastric hypo/achlorhydria due to the widely use of antisecretagous drugs as H2RBs and PPI has repeatedly been shown to increase the susceptibility to several bacterial and parasitic infections. However, the resulting susceptibility to infections has not been studied extensively, and the majority of existing studies have had a limiting retrospective observational design and have been mainly restricted to bacterial and parasitic infections only. Thus, we must be cautious about drawing broad conclusions about the use of these drugs based on the current level of evidence. The strongest evidence concerning the relationship between drug-induced hypo/achlorhydria and predisposition to infections relates to bacterial infections affecting the gastrointestinal tract and to changes in the gut microbiome. Taken into consideration the existing, though limited evidence concerning a variety of infections with different microbiological agents, it seems reasonable to conclude that the removal of gastric acid has significant clinical consequences. Consequently, this potential risk should be considered before using antisecretagous drugs and it underlines the importance of prescription for clinically appropriate indications, avoiding broad off-label use and having a prudent time-limited endpoint of prescription. Indeed, further studies are needed to evaluate the clinical consequences of impaired gastric acidity with respect to susceptibility to infections.

## Figures and Tables

**Table 1:** Infections in the gastrointestinal tract reported to be associated with gastric hypochlorhydria.

*Bacterial Infections*	*Parasitic Infections*	*Fungal Infections*
*Non-typhoid salmonellosis*	*Strongyloides*	*Candida alibicans*
*Cholera*	*Giardia*	
*Campylobacter jejuni*	*Entamoeba histolytica*	
Diarrhoeagenic *Escherichia coli*		
*Clostridium difficile*		
Shigellosis		
Listeriosis		
Brucellosis		

**Table 2:** Patient groups with reported association between use of antisecretagogues and increased risk of pneumonia.

Patient Group
Patients in ICU units
Dementia
Acute stroke
Type II diabetes mellitus
Older adults
Cirrhosis
GERD

## Abbreviations

HCl	hydrochloric acid
H2RB	histamine-2 receptor blocker
PPI	proton pump inhibitor
GERD	gastro-esophageal reflux disease
ECL	enterochromaffin-like
RR	relative risk
OR	odds ratio
CDI	*Clostridium difficile* infection
rCDI	recurrent *Clostridium difficile* infection
ICU	intensive care unit
HR	hazard ratio
SBP	spontaneous bacterial peritonitis
HE	hepatic encephalopathy
